# Identification of Clinically Relevant Fungi and *Prototheca* Species by rRNA Gene Sequencing and Multilocus PCR Coupled with Electrospray Ionization Mass Spectrometry

**DOI:** 10.1371/journal.pone.0098110

**Published:** 2014-05-16

**Authors:** Xuan Wang, Yong-Feng Fu, Rui-Ying Wang, Li Li, Ya-Hui Cao, Yan-Qiong Chen, Hua-Zhen Zhao, Qiang-Qiang Zhang, Ji-Qin Wu, Xin-Hua Weng, Xun-Jia Cheng, Li-Ping Zhu

**Affiliations:** 1 Department of Infectious Diseases, Huashan Hospital, Fudan University, Shanghai, China; 2 Department of Medical Microbiology and Parasitology, Shanghai Medical College of Fudan University, Shanghai, China; 3 Mycology Lab, Department of Dermatology, Huashan Hospital, Fudan University, Shanghai, China; University of Pittsburgh, United States of America

## Abstract

**Background:**

Multilocus PCR coupled with electrospray ionization mass spectrometry (PCR/ESI-MS) is a new strategy for pathogen identification, but information about its application in fungal identification remains sparse.

**Methods:**

One-hundred and twelve strains and isolates of clinically important fungi and *Prototheca* species were subjected to both rRNA gene sequencing and PCR/ESI-MS. Three regions of the rRNA gene were used as targets for sequencing: the 5′ end of the large subunit rRNA gene (D1/D2 region), and the internal transcribed spacers 1 and 2 (ITS1 and ITS2 regions). Microbial identification (Micro ID), acquired by combining results of phenotypic methods and rRNA gene sequencing, was used to evaluate the results of PCR/ESI-MS.

**Results:**

For identification of yeasts and filamentous fungi, combined sequencing of the three regions had the best performance (species-level identification rate of 93.8% and 81.8% respectively). The highest species-level identification rate was achieved by sequencing of D1/D2 for yeasts (92.2%) and ITS2 for filamentous fungi (75.8%). The two *Prototheca* species could be identified to species level by D1/D2 sequencing but not by ITS1 or ITS2. For the 102 strains and isolates within the coverage of PCR/ESI-MS identification, 87.3% (89/102) achieved species-level identification, 100% (89/89) of which were concordant to Micro ID on species/complex level. The species-level identification rates for yeasts and filamentous fungi were 93.9% (62/66) and 75% (27/36) respectively.

**Conclusions:**

rRNA gene sequencing provides accurate identification information, with the best results obtained by a combination of ITS1, ITS2 and D1/D2 sequencing. Our preliminary data indicated that PCR/ESI-MS method also provides a rapid and accurate identification for many clinical relevant fungi.

## Introduction

Over the past few decades, invasive fungal diseases (IFDs) have become a big challenge to public health, especially in hospitalized patients who are critically ill or immunocompromised. Despite extraordinary development in antifungal drugs during these years, the mortality of IFDs remains high [1], [2]. As there are species-specific differences in susceptibility to antifungal agents for many pathogenic fungi, rapid and accurate identification of organisms to species level is essential for the early initiation and optimal choice of antifungal therapy. Optimal treatment is of importance to reduce mortality [3], [4]. In most routine diagnostic laboratories, phenotypic identification is used, based on morphological and biochemical analysis. Technical expertise and a great deal of time are required for morphological identification, especially for some less common species. Phenotypic methods have also long been criticized for low accuracy and database limitations [5], [6].

The development of molecular approaches has greatly facilitated the identification of fungal pathogens. Many molecular identification methods have been established, such as PCR-based sequencing, restriction fragment length polymorphism (RFLP), pyrosequencing, matrix-assisted laser desorption ionization–time of flight mass spectrometry (MALDI-TOF MS), and multilocus PCR coupled with electrospray ionization mass spectrometry (PCR/ESI-MS) [7]–[9]. Among these methods, PCR amplification followed by sequencing and pairwise alignment of amplicons has been widely accepted as the “gold standard” for fungal identification. PCR-based sequencing demonstrates better specificity and accuracy compared with traditional phenotypic methods [10], [11]. Because of sufficient intra-species conservation and inter-species specificity, the 5′ end of the large subunit rRNA gene (D1/D2 region) and the internal transcribed spacer 1 and 2 (ITS1 and ITS2 regions) between 18 s and 28 s rRNA genes are most frequently used as targets for sequencing [12]–[14]. Nonetheless, this method has its limitations in the requirement for high quality and quantity of DNA samples, and the poor performance in characterizing mixtures of organisms [15].

PCR/ESI-MS which is performed on the commercialized instrument PLEX-ID*™* system (Abbott Molecular Inc., Des Plaines, IL, USA) couples multilocus PCR amplification with automated electrospray ionization mass spectrometry analysis and accurately weighs the amplicons, or mixture of amplicons to deduce the composition of A, G, C and T for each amplicon. By comparing with a database of calculated base compositions derived from sequences of reference strains, the base compositions can provide pathogen identification [16], [17]. The ability to characterize mixtures of organisms, along with its short turnaround time, and low DNA concentration requirement, makes it a promising method in fungal identification. Several studies have demonstrated its utilization in identification of microbes and diagnosis of bloodstream infections [17], [18]. It has been shown that PCR/ESI-MS had good performance in identifying clinical *Candida* isolates to species level [19], [20]. However, data regarding the ability of PCR/ESI-MS to identify other clinical relevant fungi isolates are still insufficient [21], [22].

In the present study, we used rRNA gene sequencing and PCR/ESI-MS methods, as well as traditional phenotypic methods, to identify a series of clinically relevant fungi including yeast and filamentous fungi, and two *Prototheca* species. The results of identification were compared among different methods, in an effort to provide further evidence for the performance of these methods in the identification of pathogenic fungi in clinical settings.

## Materials and Methods

### Strains and isolates

A total of 112 fungal isolates were used in this study. Thirteen of them were reference strains: *Candida albicans* (n = 1, ATCC 90028), *Candida parapsilosis* (n = 1, ATCC 22019), *Candida krusei* (n = 1, ATCC 6258) and *Aspergillus fumigatus* (n = 1, ATCC 10894) strains from the American Type Culture Collection (ATCC); *Candida dubliniensis* (n = 1, RI0482), *Cryptococcus neoformans* (n = 2, molecular type VN Ι and VN ΙΙ), and *Cryptococcus gattii* (n = 2, molecular type VG Ι and VG ΙV) strains from Centre for Infectious Diseases and Microbiology, the University of Sydney; *Aspergillus ustus* (n = 1, 35377), *Aspergillus versicolor* (n = 1, 35379), *Aspergillus flavipes* (n = 1, 33921), and *Aspergillus nidulans* (n = 1, CNCGF129) strains from Chinese Academy of Sciences. Ninety-three were clinical isolates cultured from various clinical specimens including blood, cerebrospinal fluid (CSF), sputum, stool, liver mass, peritoneal dialysis solution, bronchoalveolar lavage fluid, oral secretion, nasal secretion, lung tissue, eye, and wound. Six environmental isolates cultured from roots, leaves or soil were also included. Clinical relevant Gram-positive and Gram-negative bacteria including *Acinetobacter baumannii* (n = 1), *Escherichia coli* (n = 1), *Enterococcus faecalis* (n = 1), *Klebsiella pneumoniae* (n = 1), *Streptococcus pneumoniae* (n = 1), *Pseudomonas aeruginosa* (n = 1), *Staphylococcus aureus* (n = 1), as well as whole blood sample (n = 1) from a healthy adult were included as negative controls to assure the specificity of both molecular identification methods.

### Phenotypic identifications

The phenotypic identifications of all the isolates were performed in the clinical mycology laboratory of Huashan Hospital, Fudan University. Yeast species were identified by their colony morphology and microscopic morphology on Sabouraud dextrose agar (BD Biosciences, Cockeysville, MD, USA) and CHROMagar *Candida* plates (BD Biosciences). Biochemical test by API 20C AUX system (bioMéríeux, Marcy I'Etoile, France) was used as supplement for yeast identification. L-canavanine glycine bromothymol blue (CGB) agar was used for the differentiation of *C. neoformans* and *C. gattii* [23], [24]. Identifications of filamentous fungi were based on their colony morphology and microscopic morphology on Sabouraud dextrose agar (BD Biosciences, Cockeysville, MD, USA) and Czapek-Dox agar (Difco Laboratories, Detroit, MI, USA).

### Amplification and sequencing of the rRNA gene regions

The QIAamp DNA Mini Kit (QIAGEN, Valencia, CA, USA) was used for extraction of fungal, bacterial and human genomic DNA per the Qiagen Handbook's protocol. Three pairs of fungus-specific universal primers were used for amplification of the three rRNA gene regions (ITS1, ITS2 and D1/D2) (Table 1) [25]–[27]. PCR was performed in a total reaction volume of 25 µL containing 1.5 mM of MgCl_2_, 0.1 mM of each dNTP, 1.25 U of Taq DNA polymerase, 0.2 µM of each primer, 1× PCR buffer and 3 µL of DNA template. The PCR was performed for 35 cycles with 30 s of denaturation at 94°C, 30 s annealing at 61°C, and 1 min extension at 72°C, followed by a final extension cycle for 6 min at 72°C. The amplicons were sequenced using the ABI 3730 XL DNA Analyzer (Applied Biosystems/Hitachi, Foster City, CA). Pairwise sequence alignment was performed in the GenBank database (www.ncbi.nlm.nih.gov/BLAST/).

**Table 1:** Fungus-specific universal primers used for PCR amplification of rRNA genes.

Primer	Target gene	Direction	Primer sequence (5′→3′)
ITS-1	ITS1	Forward	TCCGTAGGTGAACCTGCGG
ITS-2		Reverse	GCTGCGTTCTTCATCGATGC
ITS-3	ITS2	Forward	GCATCGATGAAGAACGCAGC
ITS-4		Reverse	TCCTCCGCTTATTGATATGC
NL-1	D1/D2	Forward	GCATATCAATAAGCGGAGGAAAAG
NL-4		Reverse	GGTCCGTGTTTCAAGACGG

The criteria for judging the results of rRNA gene sequencing and alignment were adopted from previous studies with modification as follows [14], [28]: A strain or isolate was assigned to species level if the best matching reference species showed ≥98% homology and the next best matching reference species showed at least 0.8% less sequence homology. A strain or isolate was assigned to genus level when there was 95% to 98% homology to the best matching species, or when more than one sequence entry of several species from the same genus showed of ≥98% homology. “No identification (NI)” was defined as<95% homology with the best matching reference species, or when there was homology of >95% with multiple sequences of various genera. For each strain and isolate, a multiple rRNA gene sequencing identification result was integrated from alignment results of ITS1, ITS2 and D1/D2 as follows: species-level identification was defined when any of the three regions sequencing yielded species-level identification and results by the other regions were of the same species, genus, or NI result; genus level identification was defined when different species but the same genus were indicated by the three regions, or when one of them resulted in genus level identification while the remaining two showed NI result; NI was defined when all the three sequencings yielded NI results or when the results disagreed on genus level.

### Assignment of Micro ID

In order to provide a relative standard for further evaluation of results from PCR/ESI-MS, a microbial identification (Micro ID) for each strain and isolate in this study was predefined on the basis of results from both the phenotypic identification and rRNA gene sequencing [29]. For strains or isolates with consistent results between the phenotypic identification and rRNA gene sequencing, the Micro ID was the consensus result. For strains or isolates with inconsistent results between them, the Micro ID was defined primarily based on rRNA gene sequencing. Phenotype was considered supplemental to rRNA gene sequencing in cases where related species could not be differentiated by ITS or D1/D2 sequencing.

### PCR/ESI-MS analysis

Multilocus PCR for ESI-MS was performed with broad fungal assay plate (Abbott Molecular Inc, Des Plaines, IL, USA). There were 16 primer pairs in the kit with large subunit (LSU) rRNA, small subunit (SSU) rRNA, mitochondrial DNA (mtDNA) SSU, mtDNA cytochrome B (cyt B), and β-tubulin genes as molecular targets, among which eight targeted broad-range fungi, seven were species or genus specific, and one served as extraction control (Table 2). The following conditions were used for PCR on a 96-well Thermal Cyclers (Eppendorf, Hamburg, Germany): 95°C for 10 min, followed by 8 cycles of 95°C for 30 s, 48°C for 30 s (increasing 0.9°C each cycle), and 72°C for 30 s. The PCR was then continued for 37 additional cycles of 95°C for 15 s, 56°C for 20 s, and 72°C for 20 s. The cycle ended with a final extension of 2 min at 72°C, 99°C for 20 min, and then followed by a 4°C hold. Amplicons and mixtures of amplicons (47 samples were prepared in mixtures of 2–4 samples per reaction) were analyzed using the Abbott PLEX-ID universal biosensor platform (Abbott Molecular Inc, Des Plaines, IL, USA), which performed automated post-PCR desalting, ESI-MS signal acquisition, spectral analysis, and data reporting. Information such as the number of copies of DNA per well, the number of primers making the identification, and the base composition similarity between the reference and unknown isolates were taken into account to determine the correlation. The criteria for judgment of identification results were adopted from previous studies with minor modification as follows [21], [30]: A strain or isolate was assigned to species level when the retrieved loci showed ≥60% match with database signatures, with the best matching reference species showed ≥0.90 correlation and the next best matching reference species showed at least 0.1% less correlation at the same time. A strain or isolate was assigned to genus level when several matching species from the same genus met the criteria above. “Fungi detected-no identification can be provided” was defined as 50% to 60% database match. “No detection (ND)” was reported for specimens whose best database match was below 50% or when no fungus was detected.

**Table 2:** Primers used in PCR/ESI-MS analysis.

			Primer Sequences (5′→3′)	
Primer pair No.	Molecular target	Fungal Target	Forward	Reverse
3030	LSU rRNA	Broad/Many fungi	TGTGAAGCGGCAAAAGCTCAAATTT	TTCTCACCCTCTGTGACGGCCTGTTCC
5185	LSU rRNA	Broad/Many fungi	TGGAGTCTAACATCTATGCGAGTGTT	TCCCACAGCTATGCTCTTACTCAAATCC
3766	LSU rRNA	Broad/Many fungi	TTGTGTAGAATAGGTGGGAGCTTCGGC	TCTGACAATGTCTTCAACCCGGATC
5186	LSU rRNA	Broad/All fungi	TCATTTTTGGTAAGCAGAACTGGCGA	TGTCTAGATGAACTAACACCTTTTGTGGT
3865	mtDNA SSU	*Candida albicans*/*C. tropicalis*/*C. parapsilosis*	TGGTACAGTGGAGTATGCTGTTTAATTGGA	TCTGACGACAACAATGTAACGCCTG
3867	mtDNA SSU	*Candida glabrata*	TTCGATACCCGTGTAGTTCTAGTAGTAAAC	TCATTATTGCTAACGTACTCTTCAGGTGG
3862	mtDNA SSU	*Aspergillus spp.*	TCCAAGTACTTGACACATGCTAATCG	TCCCCTTACTTTAAGGTAGCCAAATTATC
4145	mtDNA SSU	*Crytococcus neoformans*/*bacillisporus*	TACCAAGCCAATGACGAGTAGGG	TGCTGCACATAATCTATGCTCTGGAC
5181	mtDNA SSU	Broad/*Ascomycetes*	TCCAAATTACGTGCCAGCAGTCG	TCCCTACCGTCTAGGTACCCTTTAAACC
4837	β-tubulin	*Fusarium solani*/*oxysporum*	TGCCGCGGTGCTCACTCTTTC	TGTATCGGCCGTTGCGGAAGTC
5178	SSU rRNA	Broad/Most fungi	TGCAGGCCTATGCTCGAATACATTAGCAT	TGTCCCTATTAATCATTACGGCGGTCCTA
5172	mtDNA cytB	*Mucor*/*Rhizopus*	TCCCCGTAACAATTTTCTTATTCTTCTTAGTATTAG	TGCATTGGGTTAGCTGGAATATAATTATCAGGATG
5174	SSU rRNA	*Mucor*/*Rhizopus*	TCGAAGACGATTAGATACCGTCGTAGTC	TCAGAGCCCAAAAACTTTACTTTCGCTAAG
4836	SSU rRNA	Broad/Many fungi	TCTTGGATTGACCGAAGACAAACTACTG	TCTCTAGTCGGCATCGTTTGTGGTTAAG
5187	LSU rRNA	Broad/Most fungi	TGCCGAAGTTTCCCTCAGGATAGCA	TCAAGGCCTCTAATCATTCGCTTTACCTC
4437	extraction control	Extraction control	TGACGAGTTCATGAGGGCAGGC	TCTGGCCTTTCAGCAAGTTTCCAAC

### Statistical analysis

Accuracy of identification assays was calculated as the proportion of samples correctly identified by each of the methods. Between-group comparisons were performed using chi-squared test. A p-value (*P*) of <0.05 was considered statistically significant. SPSS 19.0 (SPSS Inc., Chicago, IL, USA) was used for statistical analysis.

## Results

### Identification of reference strains by phenotypic methods and rRNA gene sequencing

Identification results of the 13 reference strains are shown in Table 3. Phenotypic methods yielded species-level identification for most of the strains, except one *C. dubliniensis* strain that was identified as its closely related species *C. albicans*, and five *Aspergillus spp.* strains that were identified to genus level. Sequencing of the three rRNA gene regions yielded species-level identification for most of the strains with a few exceptions. Two *C. gattii* strains were identified to genus level because of high homology between *C. neoformans* and *C. gattii* by sequencing of all the three regions. In addition, one *A. ustus* strain was correctly identified to species level by D1/D2 sequencing, but misidentified as other *Aspergillus spp.* that belonged to the *A. ustus* complex by either ITS1 or ITS2 sequencing.

**Table 3:** Identification of reference strains (n = 13).

	rRNA gene sequencing					
Reference strains	ITS1	ITS2	D1/D2	ITS1+ITS2+D1/D2	Phenotypic identification	Micro ID
*Candida albicans*	*C. albicans*	*C. albicans*	*C. albicans*	*C. albicans*	*C. albicans*	*C. albicans*
*Candida parapsilosis*	*Candida spp.* 1	*C. parapsilosis*	*C. parapsilosis*	*C. parapsilosis*	*C. parapsilosis*	*C. parapsilosis*
*Candida krusei*	*C. krusei*	*C. krusei*	*C. krusei*	*C. krusei*	*C. krusei*	*C. krusei*
*Candida dubliniensis*	*C. dubliniensis*	*C. dubliniensis*	*C. dubliniensis*	*C. dubliniensis*	*C. albicans*	*C. dubliniensis*
*Cryptococcus neoformans*	*C. neoformans*	*C. neoformans*	*C. neoformans*	*C. neoformans*	*C. neoformans*	*C. neoformans*
*C. neoformans*	*C. neoformans*	*C. neoformans*	*C. neoformans*	*C. neoformans*	*C. neoformans*	*C. neoformans*
*Cryptococcus gattii*	*Cryptococcus spp.* 2	*Cryptococcus spp.* 2	*Cryptococcus spp.* 2	*Cryptococcus spp.* 2	*C. gattii* 3	*C. gattii*
*C. gattii*	*Cryptococcus spp.* 2	*Cryptococcus spp.* 2	*Cryptococcus spp.* 2	*Cryptococcus spp.* 2	*C. gattii* 3	*C. gattii*
*Aspergillus fumigatus*	*A. fumigatus*	*A. fumigatus*	*A. fumigatus*	*A. fumigatus*	*Aspergillus spp.*	*A. fumigatus*
*Aspergillus nidulans*	*A. nidulans*	*A. nidulans*	*A. nidulans*	*A. nidulans*	*Aspergillus spp.*	*A. nidulans*
*Aspergillus ustus*	*Aspergillus spp.* 4	*Aspergillus spp.* 5	*A. ustus*	*A. ustus*	*Aspergillus spp.*	*A. ustus*
*Aspergillus versicolor*	*A. versicolor*	*A. versicolor*	*A. versicolor*	*A. versicolor*	*Aspergillus spp.*	*A. versicolor*
*Aspergillus flavipes*	*A. flavipes*	*A. flavipes*	*A. flavipes*	*A. flavipes*	*Aspergillus spp.*	*A. flavipes*

1 97% homology to *C. parapsilosis*,

2 *C. gattii*/*C. neoformans*,

3 *C. gattii* was differentiated from *C. neoformans* using L-canavanine glycine bromothymol blue (CGB) agar culture,

4 *Aspergillus calidoustus*/*Aspergillus pseudodeflectus*/*A. ustus*,

5 *Aspergillus insuetus*/*A. pseudodeflectus*/*A. ustus*.

### Identification of clinical and environmental isolates by phenotypic methods and rRNA gene sequencing

In general, 73.7% (73/99) of the clinical/environmental isolates were identified to species level by phenotypic methods. By sequencing of the ITS1, ITS2, and D1/D2 region, 73.7% (73/99), 79.8% (79/99), and 84.8% (84/99) were identified to species level, respectively. For the 64 yeast isolates, the highest species-level identification rate was yielded by D1/D2 sequencing (92.2%, 59/64), followed by phenotypic methods (89.1%, 57/64), ITS2 sequencing (84.4%, 54/64) and ITS1 sequencing (76.6%, 49/64). For the 33 filamentous fungal isolates, the species-level identification rate was highest by ITS2 sequencing (75.8%, 25/33), followed by ITS1 sequencing (72.7%, 24/33), D1/D2 sequencing (69.7%, 23/33), and phenotypic methods (48.5%, 16/33). A combination of ITS1 with ITS2 sequencing showed better performance than either of the single locus sequence analysis, and produced species-level identification rate of 89.1% (57/64) for yeasts and 81.8% (27/33) for filamentous fungi isolates (data not shown). In addition, two *Prototheca zopfii* isolates were identified to species level by D1/D2 sequencing, to genus level by phenotypic methods, but could not be identified by either ITS1 or ITS2 sequencing due to very low query coverage or identity (Table 4).

**Table 4:** Identification results of clinical and environmental isolates by phenotypic and different rRNA gene sequencing methods (n = 99).

Isolates	Number of isolates identified (%)														
	Phenotypic methods			ITS1 SQ			ITS2 SQ			D1/D2 SQ			ITS1+ITS2+D1/D2 SQ		
	SI	GI	NI	SI	GI	NI	SI	GI	NI	SI	GI	NI	SI	GI	NI
Yeasts (n = 64)	57	6	1	49	11	4	54	8	2	59	5	0	60	4	0
	(89.1)	(9.4)	(1.6)	(76.6)	(17.2)	(6.2)	(84.4)	(12.5)	(3.1)	(92.2)	(7.8)	(0)	(93.8)	(6.3)	(0)
Filamentous fungi (n = 33)	16	17	0	24	6	3	25	6	2	23	8	2	27	6	0
	(48.5)	(51.5)	(0)	(72.7)	(18.2)	(9.1)	(75.8)	(18.2)	(6.1)	(69.7)	(24.2)	(6.1)	(81.8)	(18.2)	(0)
Prototheca spp. (n = 2)	0	2	0	0	0	2	0	0	2	2	0	0	2	0	0
	(0)	(100)	(0)	(0)	(0)	(100)	(0)	(0)	(100)	(100)	(0)	(0)	(100)	(0)	(0)

ITS: the internal transcribed spacer; D1/D2: the 5′ end of the large subunit rRNA gene; SQ: sequencing; SI: species-level identification; GI: genus-level identification; NI: no identification.

Results of ITS1, ITS2, and D1/D2 sequencing were also integrated into a multiple rRNA gene sequencing result as defined in the methods section. When compared with results of sequencing of each single region, multiple rRNA gene sequencing yielded the best species-level identification (89.9%, 89/99), higher than ITS1 (73.7%, *P* = 0.003), ITS2 (79.8%, *P* = 0.047), and D1/D2 (84.8%, *P* = 0.285) sequencing.

Among the 99 isolates, concordant identification results on species level between rRNA gene sequencing and phenotypic methods were achieved for 62.6% (62/99) of them. Inconsistent results occurred for 37.4% (37/99) of them, including 32 isolates with inconsistent species-level results but consistent complex/genus level results, and 5 isolates with inconsistent results on genus level (Table S1).

### Micro IDs of reference strains and clinical/environmental isolates

When assigning Micro IDs for strains and isolates, both rRNA gene sequencing results and phenotypes were taken into consideration. The sequencing results were prioritized for most of the strains and isolates, with a few exceptions as follows: two strains indistinguishable between *C. neoformans* and *C. gattii* by rRNA gene sequencing were assigned to *C. gattii* by CGB culture, and two isolates indistinguishable between *Aspergillus oryzae* and *Aspergillus flavus* by rRNA gene sequencing were assigned to *A. flavus* by morphological and cultural characteristics [31]. Species-level Micro IDs were defined for most of the strains and isolates, except that Micro IDs were defined on complex or genus level for eight isolates including two *Candida metapsilosis*/*Candida orthopsilosis*/*C. parapsilosis* isoltates, one *Pichia guilliermondii*/*Pichia caribbica* isolate, one *Trichosporon domesticum*/*Trichosporon montevideense* isolate, two *A. versicolor*/*Aspergillus sydowii* isolates, one *A. flavus*/*A. oryzae* isolate and one *Ulocladium spp.* isolate.

### Assessment of PCR/ESI-MS analysis

Based on Micro IDs, 91.1% (102/112) of the strains and isolates, including 66 yeasts and 36 filamentous fungi, were within the coverage of PCR/ESI-MS identification as per the database of the broad fungal assay kit (Table S2). PCR/ESI-MS was able to identify 87.3% (89/102) of them to species level with 100% (89/89) consistency to their Micro IDs at species or species complex-level (*C. parapsilosis*, *C. metapsilosis*, and *C. orthopsilosis* in *C. parapsilosis* complex, *P. guilliermondii* and *P. caribbica* in *P. guilliermondii* complex, *A. versicolor* and *A. sydowii* in *A. versicolor* complex). The species-level identification rate for yeasts was 93.9% (62/66), higher than that of filamentous fungi which was 75% (27/36). Genus level identification was achieved for seven isolates of filamentous fungi, four of which were identified as *A. flavus*/*A. oryzae* within the *A. flavus* complex.

For the remaining 10 strains and isolates out of the coverage of the broad fungal assay kit, six were identified to species or genus level by PCR/ESI-MS, including two *C. gattii* strains as *C. neoformans*, one *Colletotrichum capsici* strain as *Colletotrichum boninense*, one *Mucor irregularis* isolate as *Mucor racemosus*, and one *Pichia anomala* isolate as *Pichia spp.*, all of which showed genus concordance to their Micro IDs.

PCR amplifications of the three regions for negative control samples all yielded negative results and PCR/ESI-MS analysis detected no fungus for each of them (data not shown).

## Discussion

The traditional phenotypic identification methods for pathogenic fungal species are based on morphological and biochemical characteristics of different species. Molecular approaches, especially sequence-based strategies which are being more and more widely used show the potential to be a powerful supplement for the phenotypic identification [11], [17], [32]. The ITS region is one of the most frequently used targets for sequence-based identification. It is located between the 18S and 28S rRNA gene and is divided into ITS1 and ITS2 regions by the 5.8S rRNA gene. Both ITS1 and ITS2 have important applications in sequence-based identification. Several studies have shown that ITS2 displayed significant interspecies variability to identify 99.7% of yeasts and 100% of molds to species level, while ITS1 produced identification accuracy of 96.8%–100% for yeasts and 100% for molds [13], [33]. Although ITS2 has been more frequently used for fungal identification, ITS1 showed greater interspecies variability than ITS2 region in another study [25]. A database specialized for ITS1 based on pairwise alignment searches (http://itsonedb.ba.itb.cnr.it:8080/ITS1/) has also been established. In our study, ITS2 showed higher discrimination ability than ITS1 for yeasts, and similar performance for filamentous fungi. Furthermore, a combination of ITS1 with ITS2 sequencing showed better performance than either single locus sequence alone. ITS1 plus ITS2 is also preferred to be an appropriate region for one of the taxonomic methods so called DNA-barcoding of fungi [34]–[36].

The D1/D2 region within the 26/28S large subunit rRNA gene is another commonly used target for identification [27], [37]. According to our study, D1/D2 sequencing yielded higher identification accuracy than either ITS1 or ITS2 for yeasts, in accordance with others [12]. Another study has shown that for species-level identification of some *Mucor spp.* such as *Mucor ramosissimus*, D1/D2 sequence analysis acquired higher identity than either ITS1 or ITS2 [13]. Unlike DNA-barcoding, which prefers using one standardized 500 to 800 bp sequence to identify species of all fungal pathogens, our results revealed the highest identification accuracy by combination of ITS1, ITS2 and D1/D2 sequencing for all the clinical relevant fungal pathogens we studied [36].

Though it has been generally accepted that PCR-based sequencing provides quicker and more accurate identification for fungi, the traditional phenotype-based identification remains indispensible. Phenotypes serve as essential supplements for some species that share high homology in rRNA gene sequences. In our study for example, *C. neoformans* and *C. gattii* were indistinguishable by rRNA gene sequencing but identified to species by CGB culture. Also two *Aspergillus spp.* isolates indistinguishable between *A. oryzae* and *A. flavus* by rRNA gene sequencing were identified as *A. favus* by morphological and cultural characteristics. Therefore, a combination of phenotypic and rRNA gene sequencing identification results would be better than any single one of them.

PCR/ESI-MS is a new strategy coupling broad-range PCR amplification to automated electrospray ionization mass spectrometry analysis for identification and detection of pathogens. Unlike MALDI-TOF MS, which performs the identification based on species-specific protein masses, PCR/ESI-MS relies on measurement of masses of nucleotides from PCR amplicons. In this study, we assessed the identification value of PCR/ESI-MS for various clinical relevant fungi and *Prototheca spp*. Regarding the species within the coverage of the broad fungal assay kit, 93.9% (62/66) of yeasts and 75% (27/36) of filamentous fungi were identified to species level by PCR/ESI-MS, results of which were 100% concordant to Micro ID on species/species complex level. One *C. dubliniensis* strain which is difficult to differentiate from *C. albicans* by conventional phenotypic methods was accurately identified by PCR/ESI-MS. For the identification of less common fungi by PCR/ESI-MS, a recent study showed that most *Rhodotorula spp.*, *Rhizopus spp.*, *Rhizomucor spp.*, *Fusarium spp.*, and *Scedosporium spp.* could be identified to genus level [21]. While two *Rhodotorula mucilaginosa*, four isolates of *Rhizopus spp.*, two isolates of *Rhizomucor spp.*, two isolates of *Fusarium spp.*, and two isolates of *Scedosporium spp.* in our study were successfully identified to species level. It was also noteworthy that in our study six out of ten strains and isolates out of the coverage of the kit could be identified to genus level concordant with Micro ID, indicating the potential usefulness of the kit for detection of emergent or unknown fungal pathogens.

The ability of PCR/ESI-MS to identify the filamentous fungi, as shown in our study, seemed to be not as satisfactory as for the yeasts. One of the possible explanations may be that the primers of ITS1 and ITS2 regions were not included in this kit [36], [38]. The ITS regions seem to have a particular value in the identification of filamentous fungi, as revealed by our results that either ITS1 or ITS2, yielded higher identification accuracy than D1/D2. Therefore, the inclusion of primers targeting ITS1 and ITS2 regions might possibly improve the performance of PCR/ESI-MS in the identification of filamentous fungi.

Although the 16 primer pairs in the broad fungal assay kit were designed to target different gene regions for the purpose of discriminating different genus and/or species, PCR/ESI-MS is still limited in its ability to distinguish among phylogenetically closely related species. As revealed by our results, PCR/ESI-MS could not distinguish species within the *C. parapsilosis* complex (*C. parapsilosis*, *C. metapsilosis*, and *C. orthopsilosis*) and *A. flavus* complex (*A. flavus* and *A. oryzae*). Moreover, *P. guilliermondii*, *C. neoformans*, and *A. versicolor* are included in the coverage of the kit while their closely related species such as *P. caribbica*, *C. gattii*, and *A. sydowii* are not, which makes it difficult to distinguish these morphologically similar species. A previous study showed that for ascomycetous yeasts, D1/D2 LSU sequences were not specific enough to identify closely related taxa, and the actin gene was a better marker in these cases [39]. Another study recommended β-tubulin locus for identification of individual species within various *Aspergillus* complexes, while ITS regions for inter complex level identification of *Aspergillus spp.* [40]. The intergenic spacer (IGS) sequence analysis could differentiate between *C. neoformans* and *C. gattii* in another study [41]. For distinction of *P. guilliermondii* and *P. caribbica*, sequencing of either ITS or D1/D2 regions is a good tool, as shown in results from our study and by others [42]. It seems that introduction of additional target gene regions, such as ITS and IGS, as well as different regions on LSU rRNA gene and β-tubulin gene, may be helpful to improve the ability of PCR/ESI-MS to differentiate among these closely related species, but may come with an increase of the cost meanwhile.

## Supporting Information

Table S1 Inconsistent results among rRNA gene sequencing and phenotypic identification.
(DOC)

Table S2 Identification results of strains and isolates within the coverage of broad fungal assay kit by PCR/ESI-MS analysis.
(DOC)
