# Supplementary material for: Identification of Clinically Relevant Fungi and Prototheca Species by rRNA Gene Sequencing and Multilocus PCR Coupled with Electrospray Ionization Mass Spectrometry
Source: PLoS One. 2014 May 16;9(5):e98110. doi: 10.1371/journal.pone.0098110 (PMC4024029; doi:10.1371/journal.pone.0098110)
Supplement: Table S1 — Inconsistent results among rRNA gene sequencing and phenotypic identification. (DOC) [file pone.0098110.s001.doc]

Table S1. Inconsistent results among rRNA gene sequencing and phenotypic identification.

| rRNA gene sequencing | | | |  |  |
| --- | --- | --- | --- | --- | --- |
| ITS1 | ITS2 | D1/D2 | ITS1+TIS2+D1/D2 | Phenotypic identification | Micro ID |
| Yeasts (n=16) | | | | | |
| *Candida haemulonii* | *C. haemulonii* | *C. haemulonii* | *C. haemulonii* | *Candida spp.* | *C. haemulonii* |
| *Candida orthopsilosis* | *C. orthopsilosis* | *Candida spp.* ^1^ | *C. orthopsilosis* | *Candida parapsilosis* | *C. orthopsilosis* |
| *Candida tropicalis* | *C. tropicalis* | *C. tropicalis* | *C. tropicalis* | *Candida rugosa* | *C. tropicalis* |
| *Pichia guilliermondii* | *P. guilliermondii* | *P. guilliermondii* | *P. guilliermondii* | *Candida. glabrata* | *P. guilliermondii* |
| *Pichia membranifaciens* | NI ^2^ | *P. membranifaciens* | *P. membranifaciens* | *Pichia kudriavzevii* | *P. membranifaciens* |
| *Pichia spp.* ^3^ | *Pichia anomala* | *P. anomala* | *P. anomala* | *C. glabrata* | *P. anomala* |
| *Pichia spp.* ^4^ | *Pichia spp.* ^4^ | *Pichia norvegensis* | *P. norvegensis* | *Candida spp.* | *P. norvegensis* |
| *Rhodotorula mucilaginosa* | *R. mucilaginosa* | *R. mucilaginosa* | *R. mucilaginosa* | *Rhodotorula spp.* | *R.mucilaginosa* |
| *R. mucilaginosa* | *R.mucilaginosa* | *R. mucilaginosa* | *R. mucilaginosa* | *Rhodotorula spp.* | *R.mucilaginosa* |
| *Trichosporon spp.* ^5^ | *Trichosporon spp.* ^6^ | *Trichosporon asahii* | *T. asahii* | *Trichosporon spp.* | *T. asahii* |
| *Trichosporon spp.* ^5^ | *T. asahii* | *T. asahii* | *T. asahii* | *Trichosporon spp.* | *T. asahii* |
| *Colletotrichum spp.* ^7^ | *Colletotrichum spp.* ^8^ | *Colletotrichum capsici* | *C. capsici* | Unidentified yeast | *C. capsici* |
| *Trichosporon spp.* ^9^ | *Trichosporon spp.* ^9^ | *Trichosporon spp.* ^9^ | *Trichosporon spp.* ^9^ | *Candida famata* | *Trichosporon spp.* ^9^ |
| *Pichia spp.* ^10^ | *Pichia spp.* ^10^ | *Pichia spp.* ^10^ | *Pichia spp.* ^10^ | *P. guilliermondii* | *Pichia spp.* ^10^ |
| *Candida spp.* ^11^ | *Candida spp.* ^12^ | *Candida spp.* ^11^ | *Candida spp.* ^11^ | *C. parapsilosis* | *Candida spp.* ^11^ |
| *Candida spp.* ^13^ | NI ^14^ | *Candida spp.* ^13^ | *Candida spp.* ^13^ | *C. parapsilosis* | *Candida spp.* ^13^ |
| Filamentous fungi (n=19) | | | | | |
| *Aspergillus spp.* ^15^ | *Aspergillus spp.* ^15^ | *Aspergillus spp.* ^16^ | *Aspergillus spp.* ^15^ | *Aspergillus flavus* | *A. flavus* |
| *Aspergillus spp.* ^15^ | *Aspergillus spp.* ^15^ | *Aspergillus spp.* ^16^ | *Aspergillus spp.* ^15^ | *A. flavus* | *A. flavus* |
| *Aspergillus versicolor* | *A. versicolor* | *A. versicolor* | *A. versicolor* | *Aspergillus spp.* | *A. versicolor* |
| *Mucor circinelloides* | *M. circinelloides* | *M. circinelloides* | *M. circinelloides* | *Mucor spp.* | *M. circinelloides* |
| *Mucor irregularis* | *M. irregularis* | *M. hiemalis* | *M. irregularis* | *Rhizomucor spp.* | *M. irregularis* |
| *Rhizopus microsporus* | *Rhizopus spp.* ^17^ | *R. microsporus* | *R. microsporus* | *Rhizopus spp.* | *R. microsporus* |
| *Rhizopus oryzae* | *R. oryzae* | *Rhizopus spp.* ^18^ | *R. oryzae* | *Mucor spp.* | *R. oryzae* |
| *R. oryzae* | *R. oryzae* | *R. oryzae* | *R. oryzae* | *Rhizopus spp.* | *R. oryzae* |
| *R. oryzae* | *R. oryzae* | *R. oryzae* | *R. oryzae* | *Rhizopus spp.* | *R. oryzae* |
| *Rhizomucor pusillus* | *R. pusillus* | *R. pusillus* | *R. pusillus* | *Mucor spp.* | *R. pusillus* |
| *Lichtheimia corymbifera* | *L. corymbifera* | *L. corymbifera* | *L. corymbifera* | *Lichtheimia spp.* | *L. corymbifera* |
| NI ^19^ | *L. corymbifera* | NI ^20^ | *L. corymbifera* | *Lichtheimia spp.* | *L. corymbifera* |
| *Paecilomyces spp.* ^21^ | *Paecilomyces variotii* | *P. variotii* | *P. variotii* | *Paecilomyces spp.* | *P. variotii* |
| *Fusarium oxysporum* | *F. oxysporum* | *F. oxysporum* | *F. oxysporum* | *Fusarium spp.* | *F. oxysporum* |
| *Fusarium solani* | *F. solani* | *F. solani* | *F. solani* | *Fusarium spp.* | *F. solani* |
| *Ulocladium spp.* ^22^ | *Ulocladium spp.* ^22^ | NI ^23^ | *Ulocladium spp.* ^22^ | *Ulocladium spp.* | *Ulocladium spp.* ^22^ |
| *Aspergillus spp.* ^15^ | NI ^24^ | *Aspergillus spp.* ^15^ | *Aspergillus spp.* ^15^ | *Aspergillus spp.* | *Aspergillus spp.* ^15^ |
| *Aspergillus spp.* ^25^ | *Aspergillus spp.* ^26^ | *Aspergillus spp.* ^26^ | *Aspergillus spp.* ^26^ | *Aspergillus spp.* | *Aspergillus spp.* ^26^ |
| NI ^27^ | NI ^28^ | *Aspergillus spp.* ^26^ | *Aspergillus spp.* ^26^ | *Aspergillus spp.* | *Aspergillus spp.* ^26^ |
| *Prototheca spp.* (n=2) | | | | | |
| NI ^29^ | NI ^30^ | *Prototheca zopfii* | *P. zopfii* | *Prototheca spp.* | *P. zopfii* |
| NI ^30^ | NI ^30^ | *P. zopfii* | *P. zopfii* | *Prototheca spp.* | *P. zopfii* |

^1^ *C. orthopsilosis*/*C. parapsilosis*, ^2^ 84% homology to *P. membranifaciens*, ^3^ 97% homology to *P. anomala*, ^4^ 97% homology to *P. norvegensis*, ^5^ *T. asahii*/*Trichosporon insectorum*/*Trichosporon faecale*, ^6^ 96% homology to *T. asahii*, ^7^ *Colletotrichum dematium*/*C. capsici*, ^8^ *Colletotrichum truncatum*/*C. capsici*, ^9^ *Trichosporon domesticum*/*Trichosporon montevideense*, ^10^ *P. guilliermondii*/*Pichia caribbica*, ^11^ *Candida metapsilosis*/*Candida orthopsilosis*/*C. parapsilosis*, ^12^ *C. metapsilosis*/*C. orthopsilosis*, ^13^ *C. metapsilosis*/*C. parapsilosis*, ^14^ 82% homology to *C. metapsilosis*, ^15^ *A. flavus*/*Aspergillus oryzae*, ^16^ *A. flavus* / *A. oryzae* /*Aspergillus parasiticus*, ^17^ 95% homology to *R. microsporus*, ^18^ *R. oryzae*/*Rhizopus delemar*, ^19^ 94% homology to *L. corymbifera*, ^20^ 71% homology to *L. corymbifera*, ^21^ 95% homology to *P. variotii*, ^22^ *Ulocladium chartarum*/*Ulocladium atrum*/*Ulocladium botrytis*, ^23^ *Alternaria alternata*/*U. chartarum*/*Montagnula dura*, ^24^ *A. flavus/A. oryzae/ F. solani*, ^25^ *A. versicolor*/*Aspergillus sydowii*/*A. flavus*, ^26^ *A. sydowii*/*A. versicolor*, ^27^ 91% homology to *A. versicolor*/*A. sydowii*/*A. flavus*, ^28^ 75% homology to *A. versicolor*/*A. sydowii*/*A. flavus*, ^29^ 42% query coverage when BLAST, ^30^ 86% homology to *P. zopfii*.
