# Supplementary material for: Identification of Clinically Relevant Fungi and Prototheca Species by rRNA Gene Sequencing and Multilocus PCR Coupled with Electrospray Ionization Mass Spectrometry
Source: PLoS One. 2014 May 16;9(5):e98110. doi: 10.1371/journal.pone.0098110 (PMC4024029; doi:10.1371/journal.pone.0098110)
Supplement: Table S2 — Identification results of strains and isolates within the coverage of broad fungal assay kit by PCR/ESI-MS analysis. (DOC) [file pone.0098110.s002.doc]

Table S2. Identification results of strains and isolates within the coverage of broad fungal assay kit by PCR/ESI-MS analysis.

|  | Micro ID | No. | PCR/ESI-MS identification | No. |
| --- | --- | --- | --- | --- |
| Yeasts (n = 66) | *Candida albicans* | 19 | *C. albicans* | 19 |
|  | *Candida dubliniensis* | 1 | *C. dubliniensis* | 1 |
|  | *Candida parapsilosis* | 9 | *C. parapsilosis* | 9 |
|  | *C. parapsilosis/ Candida metapsilosis/ Candida orthopsilosis* | 2 | *C. parapsilosis* | 2 |
|  | *C. orthopsilosis* | 1 | *C. parapsilosis* | 1 |
|  | *Candida glabrata* | 8 | *C. glabrata* | 8 |
|  | *Candida krusei* | 3 | *C. krusei* | 3 |
|  | *Candida tropicalis* | 7 | *C. tropicalis* | 7 |
|  | *Candida rugosa* | 2 | *C. rugosa* | 1 |
|  |  |  | ND | 1 |
|  | *Pichia caribbica/ Pichia guilliermondii* ^1^ | 1 | *P. guilliermondii* | 1 |
|  | *P. guilliermondii* | 1 | *P. guilliermondii* | 1 |
|  | *Pichia membranifaciens* | 1 | *P. membranifaciens* | 1 |
|  | *Pichia norvegensis* | 1 | *P. norvegensis* | 1 |
|  | *Issatchenkia terricola* | 1 | *I. terricola* | 1 |
|  | *Rhodotorula mucilaginosa* | 2 | *R. mucilaginosa* | 2 |
|  | *Clavispora lusitaniae* | 1 | *C. lusitaniae* | 1 |
|  | *Yarrowia lipolytica* | 1 | ND | 1 |
|  | *Cryptococcus neoformans* | 3 | *C. neoformans* | 3 |
|  | *Trichosporon asahii* | 2 | ND | 2 |
| Filamentous fungi (n = 36) | *Aspergillus fumigatus* | 7 | *A. fumigatus* | 6 |
|  |  |  | *Aspergillus spp.* ^2^ | 1 |
|  | *Aspergillus niger* | 2 | *A. niger* | 2 |
|  | *Aspergillus flavus/ Aspergillus oryzae* | 1 | *Aspergillus spp.* ^3^ | 1 |
|  | *A. flavus* | 3 | *Aspergillus spp.* ^3^ | 3 |
|  | *Aspergillus terreus* | 1 | *A. terreus* | 1 |
|  | *Aspergillus versicolor* | 2 | *A. versicolor* | 2 |
|  | *A. versicolor/ Aspergillus sydowii* ^4^ | 2 | *A. versicolor* | 1 |
|  |  |  | ND | 1 |
|  | *Aspergillus ustus* | 1 | *A. ustus* | 1 |
|  | *Aspergillus flavipes* | 1 | *Aspergillus spp.* ^5^ | 1 |
|  | *Aspergillus nidulans* | 1 | *A. nidulans* | 1 |
|  | *Mucor circinelloides* | 1 | *Mucor spp.* ^6^ | 1 |
|  | *Rhizopus microsporus* | 1 | *R. microsporus* | 1 |
|  | *Rhizopus oryzae* | 3 | *R. oryzae* | 3 |
|  | *Rhizomucor pusillus* | 1 | *R. pusillus* | 1 |
|  | *Lichtheimia corymbifera* | 2 | *L. corymbifera* | 1 |
|  |  |  | ND | 1 |
|  | *Penicillium marneffei* | 2 | *P. marneffei* | 2 |
|  | *Paecilomyces variotii* | 1 | *P. variotii* | 1 |
|  | *Fusarium oxysporum* | 1 | *F. oxysporum* | 1 |
|  | *Fusarium solani* | 1 | *F. solani* | 1 |
|  | *Scedosporium apiospermum* | 1 | *S. apiospermum* | 1 |
|  | *Scedosporium prolificans* | 1 | *S. prolificans* | 1 |

^1^ *P. caribbica* is out of the coverage of broad fungal assay kit, ^2^ *Aspergillus clavatus/A. ustus/A. terreus*, ^3^ *A. flavus/A. oryzae*, ^4^ *A. sydowii* is out of the coverage of broad fungal assay kit, ^5^ *Aspergillus fischeri/Aspergillus udagawae/Aspergillus clavatus*, ^6^ *Mucor racemosus/Mucor hiemalis.*
